# Social information use and collective foraging in a pursuit diving seabird

**DOI:** 10.1371/journal.pone.0222600

**Published:** 2019-09-23

**Authors:** Julian C. Evans, Colin J. Torney, Stephen C. Votier, Sasha R. X. Dall

**Affiliations:** 1 Centre for Ecology and Conservation, University of Exeter, Penryn Campus, Penryn, Cornwall, United Kingdom; 2 School of Mathematics and Statistics, University of Glasgow, Glasgow, United Kingdom; 3 Environment & Sustainability Institute, University of Exeter, Penryn Campus, Penryn, Cornwall, United Kingdom; Santa Fe Institute, UNITED STATES

## Abstract

Individuals of many species utilise social information whilst making decisions. While many studies have examined social information in making large scale decisions, there is increasing interest in the use of fine scale social cues in groups. By examining the use of these cues and how they alter behaviour, we can gain insights into the adaptive value of group behaviours. We investigated the role of social information in choosing when and where to dive in groups of socially foraging European shags. From this we aimed to determine the importance of social information in the formation of these groups. We extracted individuals’ surface trajectories and dive locations from video footage of collective foraging and used computational Bayesian methods to infer how social interactions influence diving. Examination of group spatial structure shows birds form structured aggregations with higher densities of conspecifics directly in front of and behind focal individuals. Analysis of diving behaviour reveals two distinct rates of diving, with birds over twice as likely to dive if a conspecific dived within their visual field in the immediate past. These results suggest that shag group foraging behaviour allows individuals to sense and respond to their environment more effectively by making use of social cues.

## Introduction

Many animals have been shown to use the behaviour of conspecifics to aid in their own decision making, in a wide variety of contexts [1–6]. By using such social information, individuals can make more informed decisions whilst avoiding the costs of having to gather this information themselves [7]. While social cues can be used to make large scale decisions such as where to forage or where to breed [8, 9], they can also be used when making frequent smaller scale decisions, such as when exactly an animal looks up to scan for a predator or lowers its head to feed [10]. These small moment to moment decisions can have a substantial influence on overall group behaviour. The study of these interactions can therefore provide important insights into the adaptive value of grouping behaviours.

Studying these behaviours in the wild can be challenging, especially when interactions take place in large groups of animals. However, advances in technology, automation and analytical techniques now allow the detailed recording of fine-scale interactions among individuals in the wild [11], and examination of how these can generate large-scale patterns of group motion and behaviour [12–18]. In this study we use such techniques to examine how conspecific activity might lead to collective behavioural dynamics in an understudied group foraging system: large diving rafts of European shags *Phalacrocorax aristotelis (*hereafter shags). Thus we aim to quantify the functional significance of social information use while diving in groups.

Shags are colonial, pursuit diving seabirds that forage in sandy, shelf seas at depths of up to 40 metres, mainly on small fish such as sandeels [19]. Though often seen foraging alone, shags also frequently engage in group diving behaviour, forming large flocks (hereafter referred to as “foraging rafts”). These foraging rafts consist of up to several hundred birds moving along the water surface and diving together, displaying a high degree of cohesion and alignment [20]. Similar formations can also be found across a wide range of aquatic birds such as surf scoters (*Melanitta perspicillata*) [12], Barrow’s goldeneyes (*Bucephala islandica*) [21, 22], American White Pelicans (*Pelecanus erythrorhynchos*) [23] and numerous other cormorant (*Phalcrocorax)* species [19]. The behaviours observed within foraging rafts differ from aggregations such as the compass rafts of Guanay cormorants (*Phalcrocorax bougainvillii*) [24], seabird rafts near colonies [25] or anti-predator waterfowl flocks [26, 27]. These assemblages tend to be have a more dispersed, haphazard structure, are relatively static and feature little to no foraging behaviour.

Such foraging aggregations might form simply due to collective attraction by individuals to aggregated prey, resulting in chaotic interactions among individuals and heightened competition for access to prey [28–30]. Nevertheless, many group-diving animals display a high degree of alignment and polarity [21, 22, 31, 32]. Such coordination, combined with the high dive synchronicity displayed in these groups is likely driven by substantial benefits to aggregating in a structured fashion. These advantages must also outweigh the disadvantages of diving in groups such as increased competition [33, 34], kleptoparasitism [33], predator attraction [35] and interference [36]. Possible benefits may include individual risk dilution [37], enhanced predator detection [35, 38], increased resource access [39] or improved foraging efficiency [40]. As shags have no natural predators within our study area (the Isles of Scilly, UK) and there are few species that might compete for access to the same resources, it seems likely that the primary benefits provided in this case might be related to foraging efficiency.

Social diving may improve foraging efficiency in a number of ways. Animals could gain hydrodynamic benefits by diving together, reducing energetic costs [41]. Repeated attacks might disorganise aggregations of prey, allowing an increase in individual foraging success [42]. Groups may even engage in semi-cooperative prey herding behaviour, forcing prey into areas where they can be more easily exploited [30, 43, 44]. Improved foraging efficiency might also be achieved by utilising social information from conspecifics to reduce uncertainty about access to food [4, 45, 46], which is particularly important given the substantial time and physiological requirements of diving [47–50]. Animals could potentially reduce uncertainty by using the diving and resurfacing behaviour of conspecifics within a group as cues to the distribution and behaviour of underwater prey, whilst resting on the surface [12, 22, 51, 52]. At the very least, individuals may benefit from simply copying the diving behaviour of their conspecifics and diving synchronously to capitalise on short term increases in food availability where prey occur in ephemeral patches [21, 53].

We describe the behaviour of individuals within foraging rafts and use Bayesian methods to examine if the relative position and timing of conspecific dives influence diving behaviour [17, 54, 55]. If shags are simply diving based on their own personal information, we would assume that the timing and placement of a conspecific dive will have no effect on an individual’s dive probability. Similarly, if all individuals are reacting to the presence of prey simultaneously, we would expect to see an increased probability of diving when conspecifics have recently dived in close proximity, with no requirement that these dives occur within the individuals’ field of vision. Finally, if individuals do use the diving behaviour of conspecifics to inform their own diving decisions, we would expect an individual’s probability of diving to increase when a conspecific has recently dived in their field of vision.

## Material and methods

### Data collection

Video data were collected in the Isles of Scilly (49.9361° N, 6.3228° W, UK), from a vantage point on the island of St. Martin’s (49.9631° N, 6.2836° W) from June 13th to July 8th 2013, avoiding adverse weather conditions. This location (a shallow sandy marine embayment, S1 Fig), was selected based on evidence that rafts of shags frequently foraged in this location [20] while also providing sufficient elevation (30 m) to carry out unobstructed video analysis. Footage was captured using a video camera (Sony Handycam HDR-CX190E, Tokyo, Japan) at 25 frames per second at a resolution of 1920 × 1080 pixels. During the filming of each sequence the camera position (GPS coordinates), heading relative to magnetic north (which varied depending on the position of the flock relative to the observation point), inclination of the camera (which varied depending on the distance of the flock from the observation point, ranging from 78.4° to 80.55° with a mean of 79.46° from vertical to the bottom of the camera lens) and elevation (height of tripod 1.6 m added to the height of terrain at the observation point, a total of 31.6 m) were locked in and recorded. The lower edge of the field of view was always aligned with the nearest shoreline. Upon sighting, rafts were filmed continuously until there were no birds left in frame. The number of observed rafts during a day ranged from zero to three, with several hours usually elapsing between one raft moving out of sight/disintegrating and another arriving/forming (See S2 Fig). A total of 45 rafts were recorded (ranging from 25 to 210 birds with a mean of 103 birds), of which 26 were suitable for analysis, i.e., birds positions were able to be computationally extracted from the background (as described below), which was sometimes not possible in poor lighting conditions or if sea state was rough. From each raft recording we isolated a single 60 second clip, based on when the largest number of birds were moving through the frame. While rafts often moved perpendicular to the camera’s orientation (the long axis of the body of the camera) and thus generally moved from left to right, or right to left across the camera frame, they were not always aligned with the shore (while the camera was), often heading out into more open waters (See S3 Fig for headings of rafts relative to camera frame, and S1 Fig for the relative placement of the observation point to the shoreline and the positioning of rafts observed in a previous study).

### Video processing and position extraction

**Fig 1:**
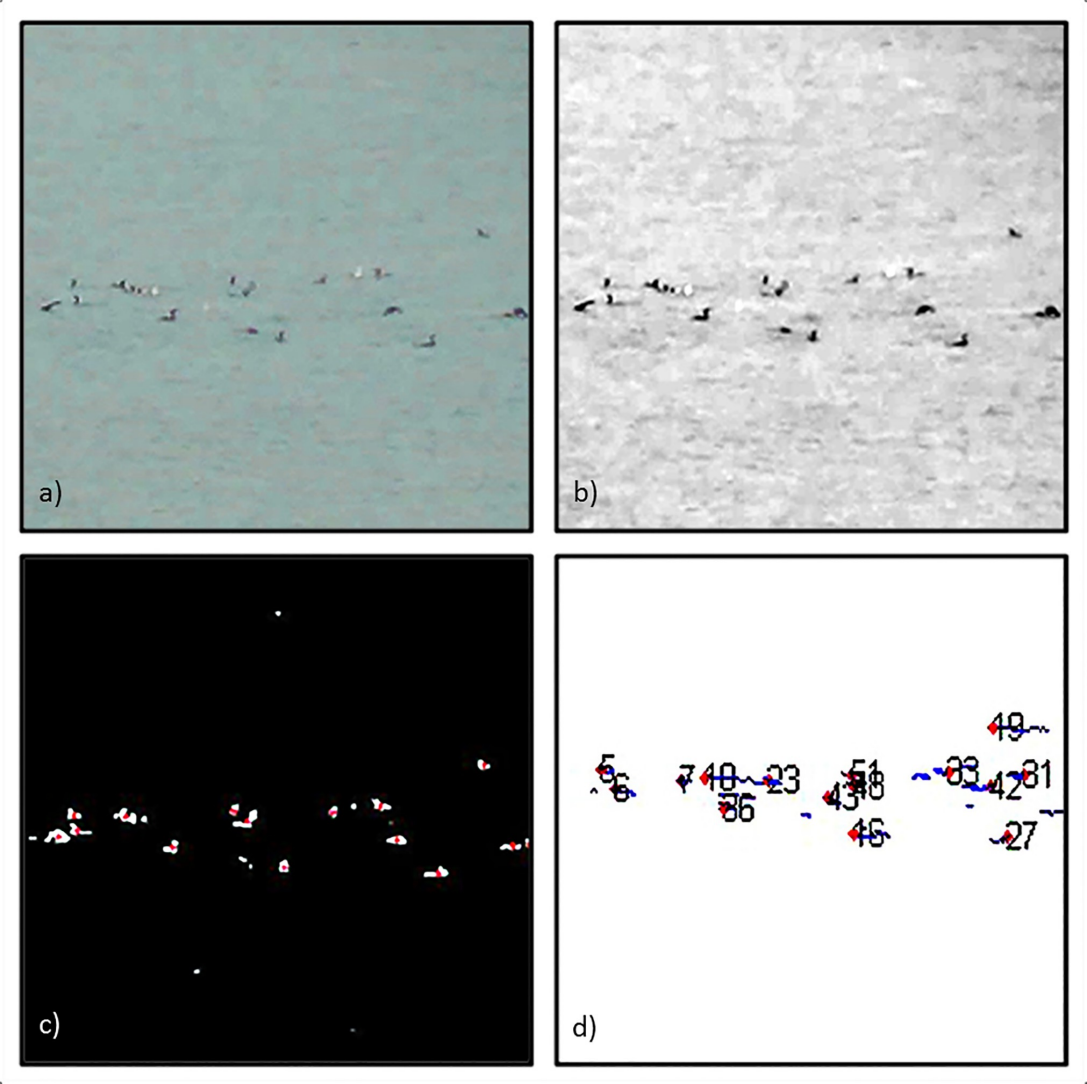
Illustration of the extraction of individual positions of group foraging European shags from video footage. A) Initial frame. B) Application of image filtering. C) Extraction of centroids coordinates. D) Creation of trajectories.

Videos were analysed frame by frame using Matlab [56]. Each frame was averaged using a selection of the preceding and successive frames in order to avoid erroneously extracting light flickering on the water (the number of frames each individual frame was averaged over was set dependant on the video, ranging from four to nine, with a mean of six frames). In each of these averaged frames birds were extracted from the background using Matlab’s image processing tools A series of image processing functions (rgb2gray, im2double, imadjust, and regionprops) were used to convert each frame into a black and white image (Fig 1B). Statistics and centroid coordinates were extracted for each individual shape present in this image (Fig 1C). These shapes were then filtered based on their areas, with shapes that were too small or too large to be birds discarded (area varied between sequences, average body size was 20 pixels). Remaining shapes’ centroid coordinates were then recorded.

### Coordinate correction and trajectory creation

Horizontal and vertical transformations were used to transform coordinates so as to correct for any perspective distortion introduced by the camera or the oblique inclination at which we filmed as detailed in the supplementary information of [12] (S4 Fig). Corrected positions from each frame were connected to create trajectories (Fig 1D). The algorithm we used predicts positions based on estimated position and velocities using linear quadratic estimation, and then calculates the distances between observations and predictions. The optimal trajectory to assign an observed position was then selected using an auction algorithm to find the nearest prediction to each observation [57]. Individuals were assumed to face their direction of movement.

Trajectories where birds appeared to be moving unrealistically fast or slow were removed (velocities of greater than 2.5 body lengths per second or less than 0.2 body lengths per second). Trajectories were checked for accuracy by reviewing back-transformed corrected positions on the original footage. Where necessary trajectories were merged or refined by deleting erroneous fixes. In the rare cases where the chosen clip included the leading edge of the flock (which generally passed rapidly out of view), individuals in this edge were excluded from the analysis as with [12]. As well as there being extremely few chosen clips where this occurred within which leading individuals were present in only a few frames, individuals on the edge of groups will experience different hydrodynamic and environmental conditions to those in the centre of the group and while interesting, this is beyond the scope of this study. Relative neighbour positions were calculated for each (non- leading edge) individual in each timestep in each of the 26 trials.

### Dive detection

Diving activity was detected by checking that trajectories that ended within a bounding-box (10 pixels smaller than the frame) met certain criteria, so as to distinguish a dive from the tracking algorithm simply losing the bird. The main check made was for an increase in area (due to the slight splash produced) followed by a rapid decrease in area over the course of two seconds prior to the end of the trajectory. The time prior to diving was also checked for sudden changes in detected heading and velocity, which could also be indicative of a dive. Instances where the algorithm accidentally detected a dive, mistimed a dive or missed a dive were manually corrected. We did not attempt to track the bird underwater, or try to link the trajectory of a surfacing individual to a previously diving individual.

### Bayesian parameter inference

To quantify the effect of a conspecific dive on a focal individual's dive probability, we modelled the dives of individual birds as a randomly occurring process that was governed by two probabilities: (1) an intrinsic probability of an individual bird diving in the absence of any social cue and (2) the probability of an individual diving in response to a conspecific dive. The detection range of shags was assumed to be defined by a fixed distance and visual angle, and responses were assumed to occur within a defined time frame.

**Fig 2:**
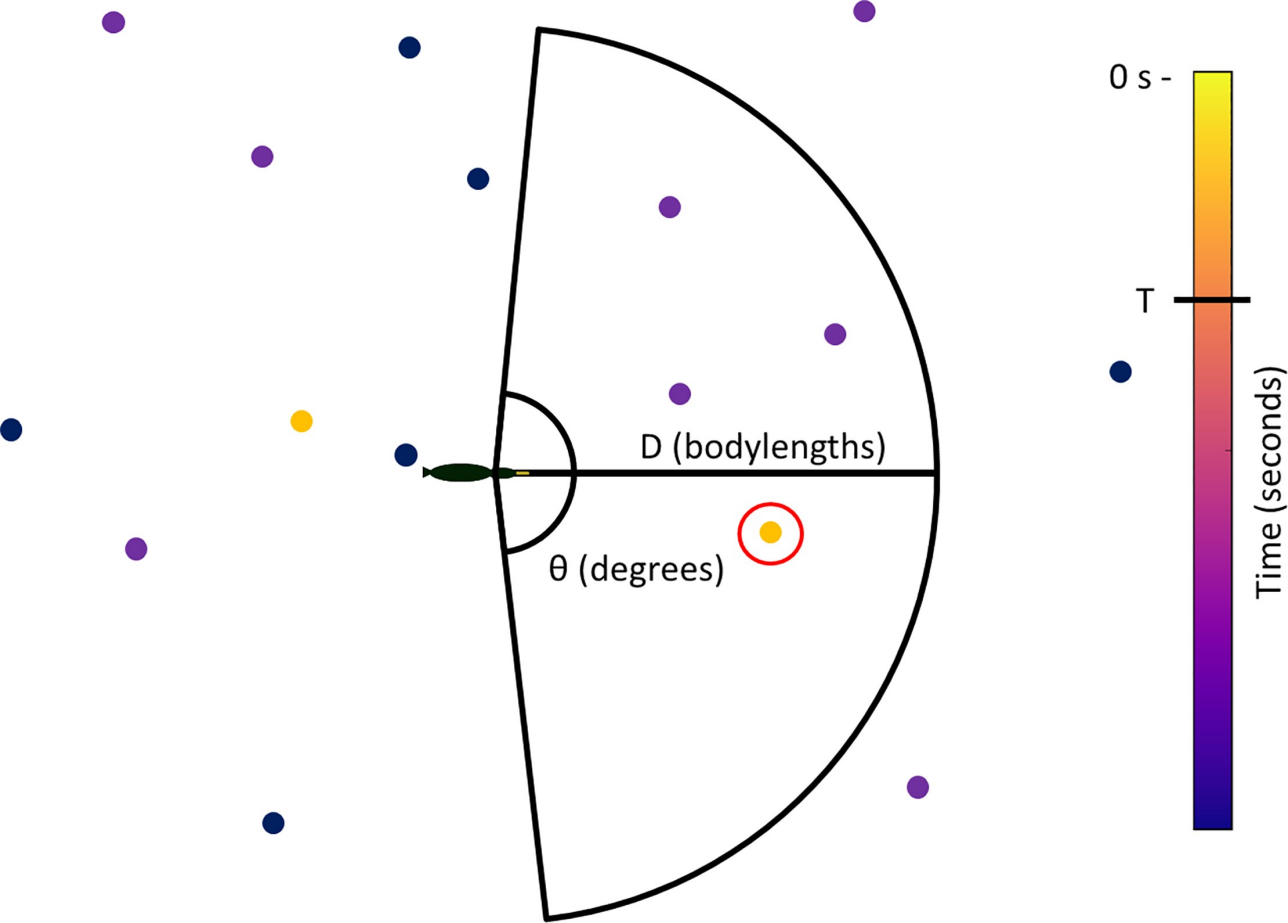
Illustration of the parameters used to determine if a focal dive is social, where the focal dive is represented by the illustration of a shag and conspecific dives are represented by coloured dots, with colour depending on how recently they had dived in time. A focal dive was defined as social if there had been a conspecific dive within the previous T seconds, at a distance less than D, and within the focal individual's visual angle θ. In the illustrated example, the dive circled in red occurred within these parameters and the focal dive would therefore be defined as social.

Trajectories were discretized into timesteps of 0.5 seconds. The diving of individual shags within each timestep was modelled as a Bernoulli process where the probability to dive was dependent on whether a neighbour had recently dived. The state of each bird, X_t_, at time t was either 0 (no dive) or 1 (a dive had begun within the interval). Specifically for the random variable X_t_ we have the following probability mass functions, P(X_t_ = 1) = p_0_ if there had been no conspecific dive within the previous T seconds (conspecific dives were modelled using their exact time rather than discretized timesteps), at a distance less than D, and within the focal individual's visual angle θ, and P(X_t_ = 1) = p_1_ if a conspecific had dived within these bounds (Fig 2).

The probabilities of parameter values conditional on the observed data were computed using Markov chain Monte Carlo methods. The Python package PyMC [58] was employed for these calculations. Priors were selected based on initial exploration of the data. Uniform priors were used over the intervals 0.5 to 2 seconds (lag time T), 0 to 100 body lengths (visual distance), 0 to 360 degrees (visual angle), 0 to 1 (dive probabilities p_0_ and p_1_). MCMC methods allowed us to sample from the posterior parameter distributions and after a burn-in period of 5000 iterations, 20000 samples were taken from these distributions which were then used to calculate mean values and their corresponding confidence intervals.

To assess the appropriateness of this model and test the predictions mentioned in the introduction, a comparison was performed between the full model and two other similar models: (1) A model which used identical parameters to the full model except for visual angle, thus meaning that birds were aware of everything in a 360 degree radius (as might be the case if all birds are simply reacting to the same stimuli) and (2) a null model featured only the intrinsic diving rate, having no social aspect and as such, no spatially clustered diving. In (1) the collective diving behaviour would be as expected if all birds were reacting to the same external stimulus. The model still retains visual distance, as this might be affected by the size of the stimulus an individual is reacting to, or the strategy used to attack a school (for example, a bird trying to attack a distant school from the side may attend to a larger area than one trying to attack from directly above a school). Model comparisons were performed using the widely applicable information criterion (WAIC) [59], a statistic that has been used in previous collective behaviour studies [60] and is more accurate than metrics based on maximum-likelihood values as it makes full use of the posterior distribution of parameter values.

### Relative positions, headings and dive plots

**Fig 3:**
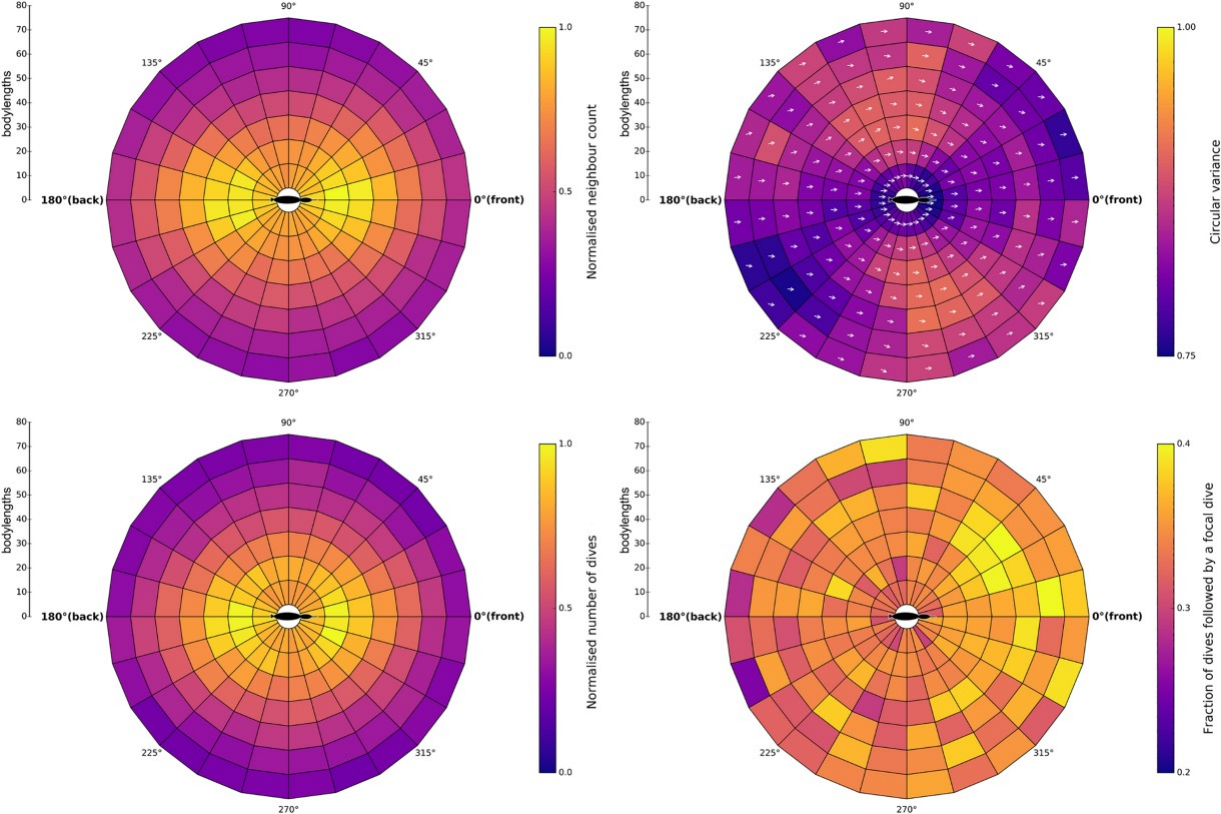
Heatmaps visualising foraging raft data. A) Relative positions of neighbours, with cell colour representing the normalised neighbour count in that area. B) Relative headings of neighbours. Arrows represent average heading in relation to relative position. Cell colour indicates the circular variance within that area. C) Positions of dives relative to other dives, with cell colour representing a normalised count of dives in that area. D) The fraction of the dives shown in C) that were followed by the focal individual diving within 2 seconds. Colour scale chosen to visualise the slight forward bias detected.

Relative neighbour positions were averaged together to produce a 2D plot of the entire dataset, giving the likelihood of a neighbour being at a position relative to a focal individual. This density plot was normalised so that the highest density was 1 (Counts in cells ranged from 0 to 76 birds depending on the size and distribution of the rafts, Fig 3A). A similar plot was created for the relative position of dive events to each other (Fig 3C). The average relative alignment of neighbours for the entire dataset, consisting of the differences in heading between a focal individual and their neighbours in each cell was also calculated, along with the variation in relative heading (circular variance) for each cell (Fig 3B). Finally, we calculated the fraction of relative dives that were "followed" by a focal bird diving in each cell (i.e., the dives that occurred within the time lag selected by Bayesian inference, Fig 3D). All relative positions were calculated and plotted using Python.

## Results

Analysis of the relative spatial distribution of shags within the raft suggested conspecifics were equally likely to occur in front and behind a focal individual and less likely to occur to the left and right of a focal individual (Fig 3A). Mean nearest neighbour distance (NND) was 9.3 ± 5.8 SD BLs (Body Lengths) and birds travelled at a mean speed of 1.1 ± 1 SD BL/s. Movement was polarised with most individuals moving in similar directions, though circular variance was high, especially to the left and right of individuals suggesting loosely aligned groups (Fig 3B, S6 Fig). Dives were highly synchronous in time (S7 Fig) and their spatial distribution closely followed that of the position of neighbours on the surface (Fig 3C), being concentrated as equally likely to occur in front or behind a focal individual. However, the number of dives that were followed by a dive from a focal individual was more heavily weighted to the front (Fig 3D), which suggests that the birds are actively following dives they have just seen occur in front of them. When we randomly assign the dive times to members of the flock, while preserving their position and orientation, the average (from 1000 randomisations) fraction of dives followed by a focal bird diving is lower, with a more uniform distribution. Therefore, the patterns we observe do not appear to be purely due to the structure of the flock (S9 Fig). Similarly, if waves of diving were passing through rafts with no social information as individuals passed over a prey patch, dives would be concentrated before (in time) and in front of or after and behind a focal bird, but this is not observed (S8 Fig). Furthermore, the speed of social transmission suggested by the Bayesian analysis (~44 Bodylengths /second = ~70 body lengths / 1.6 seconds) was faster than the average speed of birds over the water (1.1 ± 1 SD Bodylengths /second).

**Fig 4:**
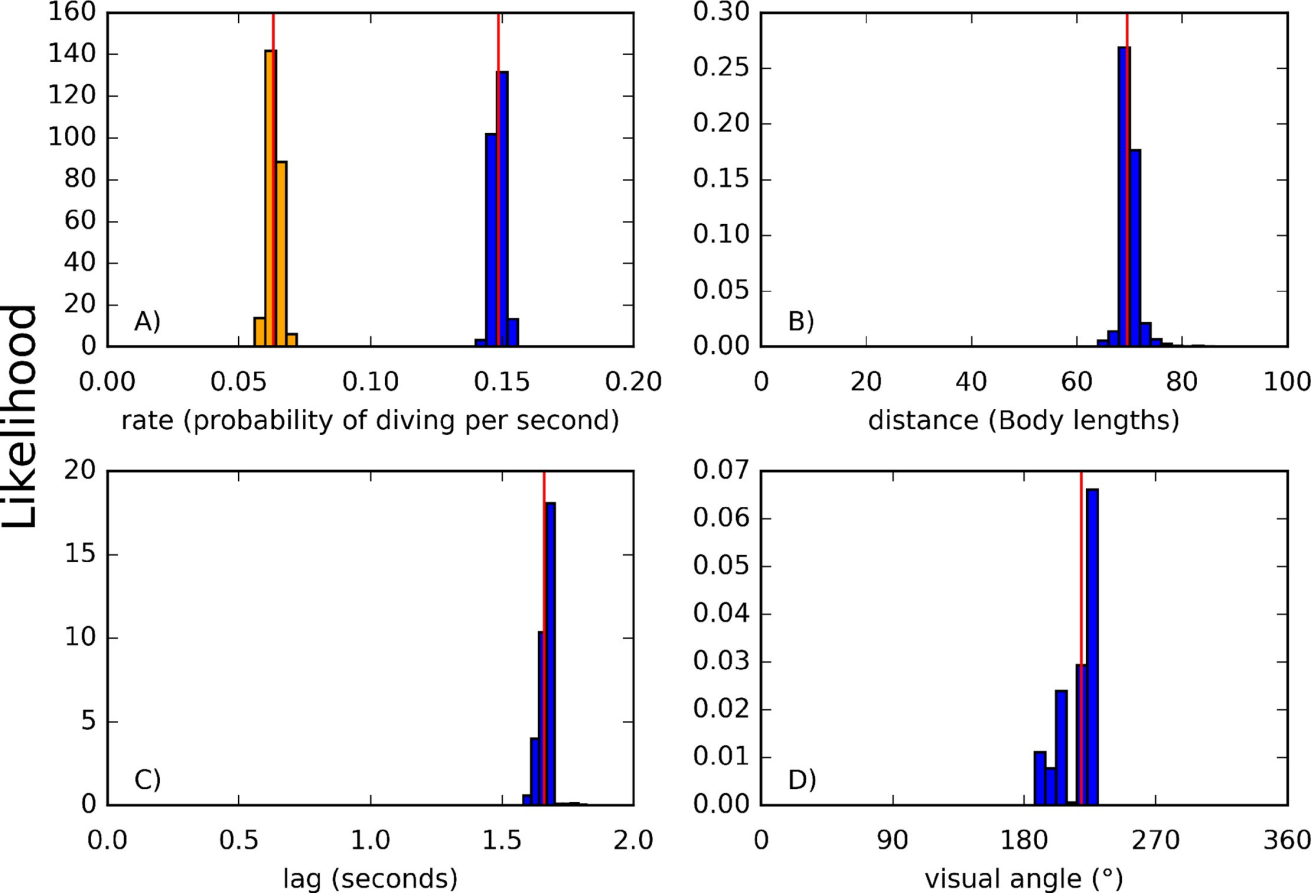
Results of Bayesian parameter inference. Bars represent estimates made during the last 20000 iterations of the Monte-Carlo Markov chain, with red lines representing the maximum likelihood estimate: A) Rates of diving, with orange representing intrinsic probability of an individual diving and blue representing probability of an individual diving after a conspecific dive has been detected. B) Maximum distance in body lengths at which a conspecific dive is detected. C) Maximum time after a conspecific dive in which that dive can be detected. D) Visual angle in front of bird in which a social dive can be detected.

Bayesian model analysis revealed the presence of two distinct diving rates (Cohen's d = 39.00, Fig 4A) with the social rate (mean = 0.1484 probability of diving per second, sd = 0.0021, 95% CI [0.1444, 0.1528]; MLE 0.1485] significantly higher than the intrinsic diving rate (mean = 0.0634 probability of diving per second, sd = 0.0022, 95% CI [0.0593, 0.0680]; MLE 0.0630). The model estimated that the probability of a bird diving increased if a conspecific had dived in the previous 1.67 seconds (Fig 4C, sd = 0.03, 95% CI [1.61,1.70]; MLE 1.66), within an arc of 216.06° (Fig 4D, sd = 11.41, 95% CI [188.49, 225.39]; MLE 218.87) centred in front of the bird, and at a distance of less than 70.28BL (Fig 4B, sd = 1.93, 95% CI [67.56, 74.74]; MLE 69.52). The estimated interaction ranges are consistent with current knowledge about the field of vision of cormorant species [52].

The model including a social diving rate dependent on a visual angle was found to be the best fitting model, outperforming the model with no visual angle (ΔWAIC = -157.80) and the null model (ΔWAIC = -898.45). These results formalise the forward bias of followed dives (Fig 3D).

## Discussion

Here we present a systematic study of the use of social cues and behavioural rules governing group diving behaviour in an air-breathing marine animal. Using automated tracking techniques and Bayesian model inference we build upon previous studies of collective behaviour of bird flocks by examining social diving dynamics [12]. The results improve our understanding of the functional significance of the common, yet understudied behaviour of diving in groups.

Shag foraging rafts were found to be relatively slower moving and less dense than other bird flocks previously studied [12]. The peak in density in front of and behind individuals indicates a high degree of following behaviour within the rafts. While this is consistent with what would be expected if shags were rafting for anti-predation benefits, this seems an unlikely explanation, due to the aforementioned lack of natural predators in the area. There may however be benefits to diluting the risk from kleptoparasites, as gulls were observed attacking shags, although such interactions were rare. It therefore seems most likely that birds are following conspecific dives because they cue prey availability or because diving synchronously confers some benefit [61].

The model which best fit our data was one in which birds were more likely to dive if a conspecific within their field of vision had recently dived. This was superior to a model in which the probability of diving was unaffected by conspecifics, as would be the case if birds were diving randomly. The full model was also a better fit than a model where a conspecific dive did not have to occur within the field of vision of the focal bird to change the probability of diving, which might be expected if all birds were diving in response to the same stimuli (e.g. presence of prey) rather than each other or using the sound of diving conspecifics as cues. Similarly, the speed of social transmission indicated by the analysis was faster than the average speed birds travelled over the water. Taken along with the forward facing visual angle suggested by the best-fitting model, these results suggest that individuals are not simply reacting to the same underwater cues as their conspecifics as they pass over a prey patch. It should be noted that while the best model suggests that conspecific dives up to 70 BL away could be detected, most recorded dives took place in relatively close proximity. Though there is nothing to suggest that shags could not detect dives at this range [52, 62], this result may be due to the lack of data at longer distances.

These results point towards shags following a strategy where individuals observe and copy conspecific dives. Copying the dives of conspecifics might allow shags to take immediate advantage of short term local prey availability, avoiding the cost of making uninformed sampling dives [63]. This would explain the short latencies between conspecific and focal dives suggested by our model. Additionally the diving behaviour of conspecifics could provide information about the distribution of prey underwater to individuals resting on the surface, which they could then utilise when they are ready to dive again themselves [52, 64]. Hence social diving will be advantageous even if assessing conspecific foraging success is difficult, which is likely here as shags generally swallow prey underwater [65]. Diving in the same area as a conspecific may also be beneficial because prey might by flushed, disorganised or fatigued by previous divers [42, 44, 62].

Further study would benefit from more details of behaviour while diving, possibly through the use of underwater cameras or acoustic imaging [42, 44, 66]. Animal mounted cameras would also be extremely useful in understanding information transfer, both underwater and on the surface [66–69]. It would be beneficial to test some of these ideas in other socially diving species, especially those that feed in different environments and on different prey [21, 22, 30–32]. This would elucidate the importance of social information use in other diving species. For example, the value of social diving cues is likely to vary dramatically with prey type and distribution. Shags mostly feed on highly motile prey (which likely has an influence on the patterns observed) but also forage on crevice dwelling demersal fish [67], which may necessitate different social foraging strategies. Feeding on sessile prey might lead to differences in the use of social information, such as the “waves” of synchronous dives observed in surf scoter flocks [12], or may instead explain the purely solitary foraging recorded for some cormorants. Recording the distribution, movements and reactions to attack of the prey species would greatly aid any future study [44].

Our results suggest that shags utilise the diving behaviour of conspecifics to inform personal dive decisions. This may be due to individuals using the surface movements and diving behaviour of neighbours as indicators of prey underwater and actively copying their decisions. This raises further questions about how social information might be useful in these types of scenario. For example, when is it best to copy others behaviour, and do all individuals have an equal probability of being copied? It could be speculated that an ability to judge the foraging success of a surfacing individual might be useful, but this would likely require more time and effort spent paying attention to the foraging behaviour of other individuals. This extra investment in gathering more detailed social information may come at the cost in missed opportunities to make dives based on personal information. Alternatively if the best strategy is simply always to copy, this level of detail might be unnecessary. We propose that increased foraging efficiency due to the ability to obtain social information from conspecifics may be one advantage of choosing to dive in a group. Our work contributes to the growing list of species for which we have evidence of social information use.

## Supporting information

S1 Video Example of tracked video. Detected dives indicated in yellow.
(MP4)

S1 Fig 95% Kernel of rafting locations observed in a previous study in relation to bathymetry. These results were used in the selection of the observation point to record collective behaviour in this study [20, 70].
(TGA)

S2 Fig Histogram of time between successive raft observations. (TGA)

S3 Fig Histogram of average heading of observed rafts relative to the camera frame, where a raft with heading 0 is moving right directly across the frame (as in S1 Vid) and a raft with heading 180 is moving left across the frame. (TGA)

S4 Fig A) Example of the calibration image used to test the corrections remove sufficient amount of distortion from the positions obtained from images (as in [12]). B) Marked grid points (red) and reconstructed grid points (blue), with the camera angled at 79° from the vertical -approximately the mean camera angle used in the field. As with Lukeman et al. we do find a slight pincushion distortion (image aberration that compresses the centre of the field) but we assume this will be negligible, particularly when coordinates are transformed into bodylengths.
(TGA)

S5 Fig Trace plots of estimates made during the Monte-Carlo Markov chain, with red lines representing the maximum likelihood estimate, calculated from the last 20000 iterations after the initial burn in period of 5000 (as indicated by the dashed line). A) Rates of diving, with orange representing intrinsic probability of an individual diving and blue representing probability of an individual diving after a conspecific dive has been detected. B) Maximum distance in body lengths at which a conspecific dive is detected. C) Maximum time after a conspecific dive in which that dive can be detected. D) Visual angle in front of bird in which a social dive can be detected. Due to the discrete cut-offs we employed in our interaction range, we observed some meta-stability in the visual angle. This represents minor fluctuations of around 10 degrees as seen in the reflected in the uncertainty intervals reported in the main text.
(PNG)

S6 Fig Histogram of relative headings of individuals in difference in degrees from heading of focal bird, where 0 would be exactly the same heading as a focal individual and 180/-180 the opposite direction. (TGA)

S7 Fig Histogram of intervals between successive dives in seconds. (TGA)

S8 Fig Illustration of relative time from dives against distance ahead/behind focal dives, where negative time is before a focal dive and negative distance. If waves of diving were occurring (as might occur if individuals were reacting as the passed over the same concentration of prey underwater) we would expect to see a concentration of dives at a positive distance and negative time (before and in front of focal dive). The speed of social transmission suggested by the Bayesian analysis (~44 Bodylengths /second = ~70 body lengths / 1.6 seconds) is also faster than the average speed of birds over the water (1.1 ± 1 SD Bodylengths /second). The asymmetry observed in Fig 2D is not observed here due to the difference in plotting scales.
(TGA)

S9 Fig The relative positions of “social” dives (As in Fig 2D) after the observed dive times have been randomly assigned to members of a flock whilst preserving their position and orientation, 1000 times. Cell colour shows the average fraction of dives that were followed by a focal bird diving within 2 seconds in each cell. Note the different colour scale due to the lower fraction of dives meeting this criterion. The lower numbers of “social” dives and more uniform distributions of dives in this figure suggest that group structure is not influencing the increased likelihood of individuals copying dives occurring in front of themselves.
(TIF)

## References

[1] Worden BD, Papaj DR. Flower choice copying in bumblebees. Biology Letters. 2005;1(4):504–7. 10.1098/rsbl.2005.0368 17148244 PMC1626359

[2] Valone T. From eavesdropping on performance to copying the behavior of others: a review of public information use. Behavioral Ecology and Sociobiology. 2007;62(1):1–14. 10.1007/s00265-007-0439-6

[3] Dall SRX, Giraldeau L-A, Olsson O, McNamara JM, Stephens DW. Information and its use by animals in evolutionary ecology. Trends in Ecology and Evolution. 2005;20(4):187–93. 10.1016/j.tree.2005.01.010 16701367

[4] Evans JC, Votier SC, Dall SRX. Information use in colonial living. Biological Reviews. 2015:1–16. 10.1111/brv.12188 25882618

[5] Godin J-GJ, Herdman EJ, Dugatkin LA. Social influences on female mate choice in the guppy, Poecilia reticulata: generalized and repeatable trait-copying behaviour. Animal Behaviour. 2005;69(4):999–1005.

[6] Mery F, Varela SA, Danchin É, Blanchet S, Parejo D, Coolen I, et al Public versus personal information for mate copying in an invertebrate. Current Biology. 2009;19(9):730–4. 10.1016/j.cub.2009.02.064 19361993

[7] Webster M, Laland K. Social learning strategies and predation risk: minnows copy only when using private information would be costly. Proceedings of the Royal Society of London B: Biological Sciences. 2008;275(1653):2869–76. 10.1098/rspb.2008.0817 PMC2605836 18755676

[8] Boulinier T, McCoy KD, Yoccoz NG, Gasparini J, Tveraa T. Public information affects breeding dispersal in a colonial bird: kittiwakes cue on neighbours. Biology Letters. 2008;4(5):538–40. 10.1098/rsbl.2008.0291 18647711 PMC2610090

[9] Jones TB, Patrick SC, Arnould JP, Rodríguez-Malagón MA, Wells MR, Green JA. Evidence of sociality in the timing and location of foraging in a colonial seabird. Biology letters. 2018;14(7):20180214 10.1098/rsbl.2018.0214 29997186 PMC6083229

[10] Beauchamp G. Function and structure of vigilance in a gregarious species exposed to threats from predators and conspecifics. Animal behaviour. 2016;116:195–201.

[11] Dell AI, Bender JA, Branson K, Couzin ID, de Polavieja GG, Noldus LPJJ, et al Automated image-based tracking and its application in ecology. Trends in ecology & evolution. 2014;29(7):417–28. 10.1016/j.tree.2014.05.004. 24908439

[12] Lukeman R, Li Y-X, Edelstein-Keshet L, Simon AL. Inferring individual rules from collective behavior. Proceedings of the National Academy of Sciences of the United States of America. 2010;107(28):12576–80. 10.1073/pnas.1001763107 20616032 PMC2906562

[13] Buhl J, Sword GA, Clissold FJ, Simpson SJ. Group structure in locust migratory bands. Behavioral ecology and sociobiology. 2011;65(2):265–73.

[14] Strandburg-Peshkin A, Farine DR, Couzin ID, Crofoot MC. Shared decision-making drives collective movement in wild baboons. Science. 2015;348(6241):1358–61. 10.1126/science.aaa5099 26089514 PMC4801504

[15] Ramos A, Petit O, Longour P, Pasquaretta C, Sueur C. Collective decision making during group movements in European bison, Bison bonasus. Animal Behaviour. 2015;109:149–60.

[16] Ballerini M, Cabibbo N, Candelier R, Cavagna A, Cisbani E, Giardina I, et al Interaction ruling animal collective behavior depends on topological rather than metric distance: Evidence from a field study. Proceedings of the national academy of sciences. 2008;105(4):1232–7. 10.1073/pnas.0711437105 PMC2234121 18227508

[17] Mann RP, Perna A, Strömbom D, Garnett R, Herbert-Read JE, Sumpter DJ, et al Multi-scale inference of interaction rules in animal groups using Bayesian model selection. 2013. 10.1371/journal.pcbi.1002961 PMC3605063 23555206

[18] Herbert-Read JE, Perna A, Mann RP, Schaerf TM, Sumpter DJ, Ward AJ. Inferring the rules of interaction of shoaling fish. Proceedings of the National Academy of Sciences. 2011;108(46):18726–31. 10.1073/pnas.1109355108 PMC3219133 22065759

[19] Nelson B. Pelicans, Cormorants and Their Relatives: *Pelecanidae*, *Sulidae*, *Phalacrocoracidae*, *Anhingidae*, *Fregatidae*, *Phaethontidae*. Oxford: Oxford University Press; 2005.

[20] Evans JC, Dall SRX, Bolton M, Owen E, Votier SC. Social foraging European shags: GPS tracking reveals birds from neighbouring colonies have shared foraging grounds. Journal of Ornithology. 2016;157(1):23–32. 10.1007/s10336-015-1241-2

[21] Schenkeveld LE, Ydenberg RC. Synchronous diving by surf scoter flocks. Canadian Journal of Zoology. 1985;63(11):2516–9. 10.1139/z85-372

[22] Beauchamp G. Diving behavior in surf scoters and Barrow's goldeneyes. The Auk. 1992:819–27.

[23] Anderson JGT. Foraging Behavior of the American White Pelican (Pelecanus erythrorhyncos) in Western Nevada. Colonial Waterbirds. 1991;14(2):166–72. 10.2307/1521506

[24] Weimerskirch H, Bertrand S, Silva J, Marques JC, Goya E. Use of Social Information in Seabirds: Compass Rafts Indicate the Heading of Food Patches. PLoS ONE. 2010;5(3):e9928 10.1371/journal.pone.0009928 20360959 PMC2847911

[25] Wilson LJ, McSorley CA, Gray CM, Dean BJ, Dunn TE, Webb A, et al Radio-telemetry as a tool to define protected areas for seabirds in the marine environment. Biological Conservation. 2009;142(8):1808–17. 10.1016/j.biocon.2009.03.019.

[26] Fox A, Green A, Hughes B, Hilton G. Rafting as an antipredator response of wintering White-headed duck Oxyura leucocephala. Wildfowl. 1994;45(45):232–41.

[27] Fox A, Mitchell C. Rafting behaviour and predator disturbance to Steller's Eiders *Polysticta stelleri* in northern Norway. Journal für Ornithologie. 1997;138(1):103–9. 10.1007/bf01651656

[28] Barnard C, Thompson D, Stephens H. Time budgets, feeding efficiency and flock dynamics in mixed species flocks of lapwings, golden plovers and gulls. Behaviour. 1982:44–69.

[29] Hoffman W, Heinemann D, Wiens JA. The ecology of seabird feeding flocks in Alaska. The Auk. 1981;98(3):437–56.

[30] Battley PF, Poot M, Wiersma P, Gordon C, Ntiamoa-Baidu Y, Piersma T. Social foraging by waterbirds in shallow coastal lagoons in Ghana. Waterbirds. 2003;26(1):26–34.

[31] Berlincourt M, Arnould JPY. At-Sea Associations in Foraging Little Penguins. PLoS ONE. 2014;9(8):e105065 10.1371/journal.pone.0105065 25119718 PMC4132066

[32] Takahashi A, Sato K, Nishikawa J, Watanuki Y, Naito Y. Synchronous diving behavior of Adélie penguins. Journal of Ethology. 2004;22(1):5–11. 10.1007/s10164-003-0111-1

[33] Ranta E, Rita H, Lindstrom K. Competition versus cooperation: success of individuals foraging alone and in groups. American Naturalist. 1993:42–58. 10.1086/285528 19425970

[34] Foster SA. Group foraging by a coral reef fish: a mechanism for gaining access to defended resources. Animal Behaviour. 1985;33(3):782–92. 10.1016/S0003-3472(85)80011-7.

[35] Cresswell W. Flocking is an effective anti-predation strategy in redshanks, *Tringa totanus*. Animal Behaviour. 1994;47(2):433–42.

[36] Ruxton G. Foraging in flocks: non-spatial models may neglect important costs. Ecological Modelling. 1995;82(3):277–85.

[37] Roberts G. Why individual vigilance declines as group size increases. Animal Behaviour. 1996;51(5):1077–86.

[38] Beauchamp G. Should vigilance always decrease with group size? Behavioral Ecology and Sociobiology. 2001;51(1):47–52.

[39] Dall SR, Wright J. Rich pickings near large communal roosts favor ‘gang’foraging by juvenile common ravens, Corvus corax. PLoS ONE. 2009;4(2):e4530 10.1371/journal.pone.0004530 19240813 PMC2646834

[40] Götmark F, Winkler DW, Andersson M. Flock-feeding on fish schools increases individual success in gulls. Nature. 1986;319(6054):589–91. 10.1038/319589a0 3945345

[41] Noren SR, Biedenbach G, Edwards EF. Ontogeny of swim performance and mechanics in bottlenose dolphins (Tursiops truncatus). Journal of Experimental Biology. 2006;209(23):4724–31. 17114405 10.1242/jeb.02566

[42] Thiebault A, Semeria M, Lett C, Tremblay Y. How to capture fish in a school? Effect of successive predator attacks on seabird feeding success. Journal of Animal Ecology. 2016;85(1):157–67. 10.1111/1365-2656.12455 26768335

[43] Benoit-Bird KJ, Au WW. Cooperative prey herding by the pelagic dolphin, Stenella longirostris. The Journal of the Acoustical Society of America. 2009;125(1):125–37. 10.1121/1.2967480 19173400

[44] Handegard Nils O, Boswell Kevin M, Ioannou Christos C, Leblanc Simon P, Tjøstheim Dag B, Couzin Iain D. The Dynamics of Coordinated Group Hunting and Collective Information Transfer among Schooling Prey. Current Biology. 2012;22(13):1213–7. 10.1016/j.cub.2012.04.050 22683262

[45] Templeton JJ, Giraldeau L-A. Patch assessment in foraging flocks of European starlings: evidence for the use of public information. Behavioral Ecology. 1995;6(1):65–72.

[46] Dermody BJ, Tanner CJ, Jackson AL. The Evolutionary Pathway to Obligate Scavenging in Gyps Vultures. PLoS ONE. 2011;6(9):e24635 10.1371/journal.pone.0024635 21931786 PMC3169611

[47] Enstipp MR, Jones DR, Lorentsen S-H, Grémillet D. Energetic costs of diving and prey-capture capabilities in cormorants and shags (Phalacrocoracidae) underline their unique adaptation to the aquatic environment. Journal of Ornithology. 2007;148(2):593–600.

[48] Wilson RP, Hustler K, Ryan PG, Burger AE, Noldeke EC. Diving Birds in Cold Water: Do Archimedes and Boyle Determine Energetic Costs? The American Naturalist. 1992;140(2):179–200. 10.2307/2462606

[49] Williams TM, Davis R, Fuiman L, Francis J, Le B, Horning M, et al Sink or swim: strategies for cost-efficient diving by marine mammals. Science. 2000;288(5463):133–6. 10.1126/science.288.5463.133 10753116

[50] Williams TM, Fuiman LA, Horning M, Davis RW. The cost of foraging by a marine predator, the Weddell seal Leptonychotes weddellii: pricing by the stroke. Journal of Experimental Biology. 2004;207(6):973–82. 10.1242/jeb.00822 14766956

[51] Stempniewicz L, Darecki M, Trudnowska E, Błachowiak-Samołyk K, Boehnke R, Jakubas D, et al Visual prey availability and distribution of foraging little auks (Alle alle) in the shelf waters of West Spitsbergen. Polar Biology. 2013;36(7):949–55. 10.1007/s00300-013-1318-4

[52] White CR, Day N, Butler PJ, Martin GR. Vision and Foraging in Cormorants: More like Herons than Hawks? PLoS ONE. 2007;2(7):e639 10.1371/journal.pone.0000639 17653266 PMC1919429

[53] Brumm H, Teschke I. Juvenile Galápagos Pelicans Increase Their Foraging Success by Copying Adult Behaviour. PLoS ONE. 2012;7(12):e51881 10.1371/journal.pone.0051881 23251646 PMC3522586

[54] Strandburg-Peshkin A, Twomey CR, Bode NWF, Kao AB, Katz Y, Ioannou CC, et al Visual sensory networks and effective information transfer in animal groups. Current Biology. 2013;23(17):R709–R11. 10.1016/j.cub.2013.07.059 24028946 PMC4780851

[55] Bode NW, Wagoum AUK, Codling EA. Information use by humans during dynamic route choice in virtual crowd evacuations. Royal Society Open Science. 2015;2(1):140410 10.1098/rsos.140410 26064589 PMC4448793

[56] MATLAB. R2015a. Natick, Massachusetts: The MathWorks Inc.; 2015.

[57] Bertsekas D. The Auction Algorithm for Assignment and Other Network Flow Problems. New Trends in Systems Theory. Progress in Systems and Control Theory. 7: Birkhäuser Boston; 1991 p. 105–12.

[58] Patil A, Huard D, Fonnesbeck CJ. PyMC: Bayesian stochastic modelling in Python. Journal of statistical software. 2010;35(4):1 21603108 PMC3097064

[59] Watanabe S. Asymptotic equivalence of Bayes cross validation and widely applicable information criterion in singular learning theory. Journal of Machine Learning Research. 2010;11(Dec):3571–94.

[60] Ferguson EA, Matthiopoulos J, Insall RH, Husmeier D. Inference of the drivers of collective movement in two cell types: Dictyostelium and melanoma. Journal of The Royal Society Interface. 2016;13(123):20160695. 10.1098/rsif.2016.0695 PMC5095226 27798280

[61] Galef BG Jr, Giraldeau L-A. Social influences on foraging in vertebrates: causal mechanisms and adaptive functions. Animal Behaviour. 2001;61(1):3–15. 10.1006/anbe.2000.1557 11170692

[62] White CR, Butler PJ, GrÉMillet D, Martin GR. Behavioural strategies of cormorants (Phalacrocoracidae) foraging under challenging light conditions. Ibis. 2008;150:231–9. 10.1111/j.1474-919X.2008.00837.x

[63] Krebs JR, Inman AJ. Learning and Foraging: Individuals, Groups, and Populations. The American Naturalist. 1992;140(ArticleType: research-article / Issue Title: Supplement: Behavioral Mechanisms in Evolutionary Ecology / Full publication date: Nov., 1992 / Copyright 1992 The University of Chicago):S63-S84. 10.2307/2462354 19426027

[64] Machovsky-Capuska GE, Howland HC, Raubenheimer D, Vaughn-Hirshorn R, Würsig B, Hauber ME, et al Visual accommodation and active pursuit of prey underwater in a plunge-diving bird: the Australasian gannet. Proceedings of the Royal Society B: Biological Sciences. 2012;279(1745):4118–25. 10.1098/rspb.2012.1519 22874749 PMC3441088

[65] Rands SA, Cowlishaw G, Pettifor RA, Rowcliffe JM, Johnstone RA. Spontaneous emergence of leaders and followers in foraging pairs. Nature. 2003;423(6938):432–4. http://www.nature.com/nature/journal/v423/n6938/suppinfo/nature01630_S1.html. 10.1038/nature01630 12761547

[66] Takahashi A, Sato K, Naito Y, Dunn M, Trathan P, Croxall J. Penguin–mounted cameras glimpse underwater group behaviour. Proceedings of the Royal Society of London Series B: Biological Sciences. 2004;271(Suppl 5):S281–S2. 15503994 10.1098/rsbl.2004.0182 PMC1810073

[67] Watanuki Y, Daunt F, Takahashi A, Newell M, Wanless S, Sato K, et al Microhabitat use and prey capture of a bottom-feeding top predator, the European Shag, shown by camera loggers. Marine Ecology Progress Series. 2008;356:283–93.

[68] Votier SC, Bicknell A, Cox SL, Scales KL, Patrick SC. A bird’s eye view of discard reforms: bird-borne cameras reveal seabird/fishery interactions. PLoS ONE. 2013;8(3):e57376 10.1371/journal.pone.0057376 23483906 PMC3590202

[69] Thiebault A, Mullers R, Pistorius P, Meza-Torres MA, Dubroca L, Green D, et al From colony to first patch: Processes of prey searching and social information in Cape Gannets. The Auk. 2014;131(4):595–609. 10.1642/auk-13-209.1

[70] HydroSpatial One—Gridded Bathymetry. Seazone Solutions; 2015.

