# Supplementary figures and images for: Social information use and collective foraging in a pursuit diving seabird
Source: PLoS One. 2019 Sep 23;14(9):e0222600. doi: 10.1371/journal.pone.0222600 (PMC6756525; doi:10.1371/journal.pone.0222600)

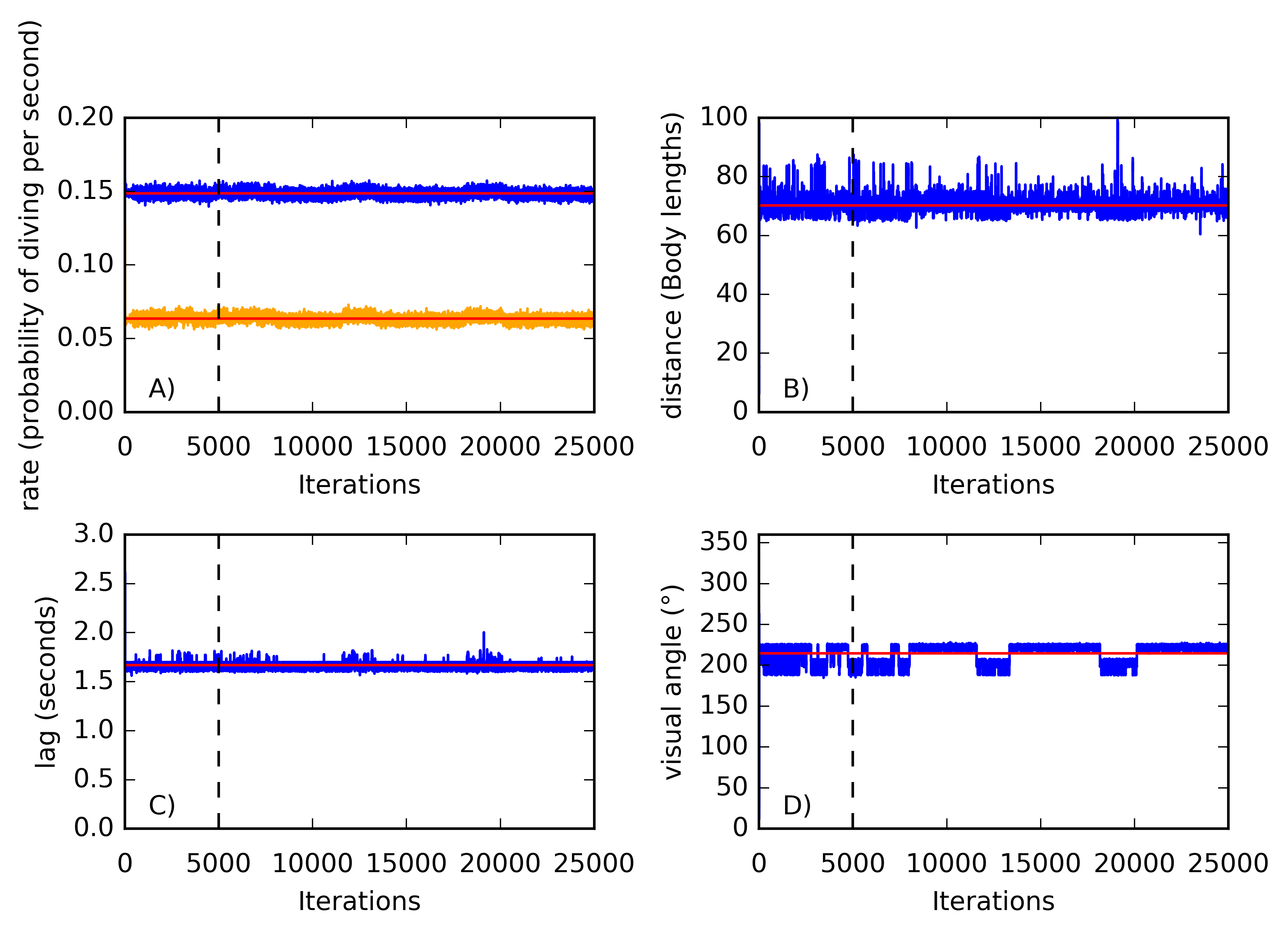

Supplement: S5 Fig — A) Rates of diving, with orange representing intrinsic probability of an individual diving and blue representing probability of an individual diving after a conspecific dive has been detected. B) Maximum distance in body lengths at which a conspecific dive is detected. C) Maximum time after a conspecific dive in which that dive can be detected. D) Visual angle in front of bird in which a social dive can be detected. Due to the discrete cut-offs we employed in our interaction range, we observed some meta-stability in the visual angle. This represents minor fluctuations of around 10 degrees as seen in the reflected in the uncertainty intervals reported in the main text. (PNG) [file pone.0222600.s006.png]

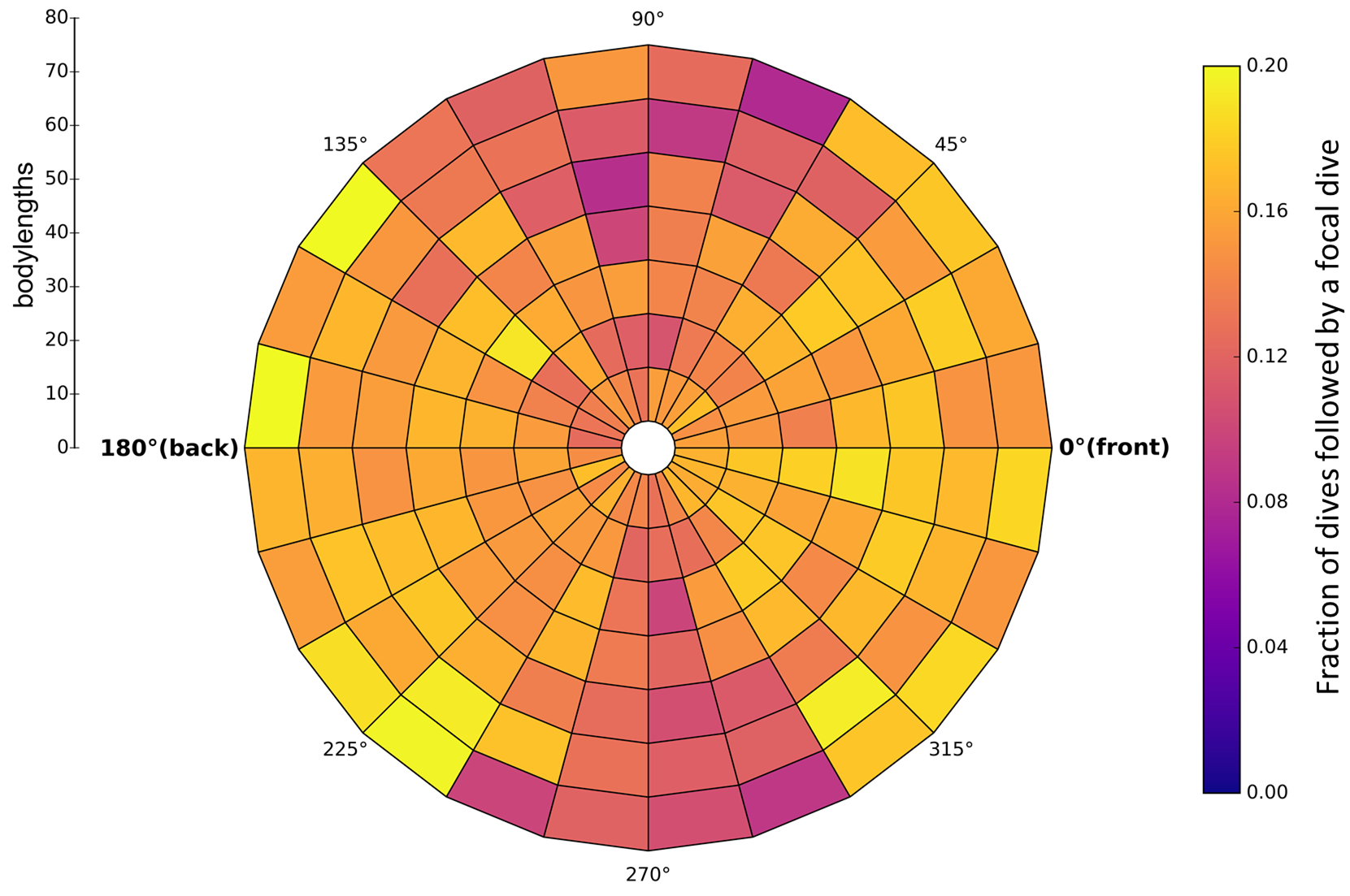

Supplement: S9 Fig — Cell colour shows the average fraction of dives that were followed by a focal bird diving within 2 seconds in each cell. Note the different colour scale due to the lower fraction of dives meeting this criterion. The lower numbers of “social” dives and more uniform distributions of dives in this figure suggest that group structure is not influencing the increased likelihood of individuals copying dives occurring in front of themselves. (TIF) [file pone.0222600.s010.tif]
